# Are Sleep Aids Associated with the Severity of Attention Deficit Hyperactivity Disorder Symptoms in Adults Screened for Insomnia? A Cross-Sectional Study

**DOI:** 10.3390/jcm13061682

**Published:** 2024-03-14

**Authors:** Omar Gammoh, Abdelrahim Alqudah, Esam Qnais, Alaa A. A. Aljabali, Ammena Y. Binsaleh, Sireen Abdul Rahim Shilbayeh

**Affiliations:** 1 Department of Clinical Pharmacy and Pharmacy Practice, Faculty of Pharmacy, Yarmouk University, Irbid 21163, Jordan; 2 Department of Clinical Pharmacy and Pharmacy Practice, Faculty of Pharmaceutical Sciences, The Hashemite University, Zarqa 13133, Jordan; abdelrahim@hu.edu.jo; 3 Department of Biology and Biotechnology, Faculty of Science, The Hashemite University, Zarqa 13133, Jordan; esamqn@hu.edu.jo; 4 Department of Pharmaceutics and Pharmaceutical Technology, Faculty of Pharmacy, Yarmouk University, Irbid 21163, Jordan; alaaj@yu.edu.jo; 5 Department of Pharmacy Practice, College of Pharmacy, Princess Nourah Bint Abdulrahman University, P.O. Box 84428, Riyadh 11671, Saudi Arabia; aysaleh@pnu.edu.sa (A.Y.B.); ssabdulrahim@pnu.edu.sa (S.A.R.S.)

**Keywords:** Attention Deficit Hyperactivity Disorder (ADHD), insomnia, sleep aids, Adult ADHD Self-Report Scale

## Abstract

(1) **Background**: Attention Deficit Hyperactivity Disorder (ADHD)-like symptoms and insomnia are closely related. The present study examined whether the use of different sleep aids was related to severe ADHD-like symptoms in Jordanian adults screened for insomnia. (2) **Methods**: This cross-sectional study used predefined inclusion criteria. The severity of ADHD was assessed using the validated Arabic version of the Adult ADHD Self-Report Scale. (3) **Results**: Data were analyzed from 244 subjects who met the inclusion criteria for severe insomnia, of which 147 (65.3%) reported not using any sleep aid, 50 (22.3%) reported using homeopathy remedies as sleep aids, and 41 (18.3%) reported using over-the-counter antihistamines as sleep aids. Regression analysis revealed that the use of such sleep aids—namely, “homeopathy herbal remedies” and “over-the-counter antihistamines”—was not associated (*p* > 0.05) with ADHD-like symptoms. However, “age above 31 years old” was significantly associated (B = −3.95, t = −2.32, *p* = 0.002) with lower ADHD severity, while the “diagnosis with chronic diseases” was significantly associated (B = 4.15, t = 1.99, *p* = 0.04) with higher ADHD severity. (4) **Conclusions**: Sleep aids are not associated with ADHD-like symptoms in adults. More research is required to uncover the risk factors for adult ADHD, especially insomnia.

## 1. Introduction

Attention Deficit Hyperactivity Disorder (ADHD), a complex neurodevelopmental condition, has been a focal point for researchers and clinicians for the past 30 years. This novel research draws upon extensive expertise to explore recent scientific findings on ADHD, highlighting the intricate interplay of genetic, environmental, and neurological factors in its manifestation. Globally, adult ADHD prevalence ranges from 2.5% to 4.4%, with the National Institute of Mental Health (NIMH) reporting an overall prevalence of 4.4%, with higher rates in males (5.4%) than females (3.2%) [1]. A 2021 global meta-analysis reported a prevalence of 2.58% for persistent adult ADHD and 6.76% for symptomatic adult ADHD [2]. Also, adult ADHD is a significant burden in different countries; for example, adult ADHD prevails in 5.2% of the population in the United States, 6% in Northern Ireland, 1.8% in China, and 2.9% in Canada [3,4].

Adult ADHD can exert a notable influence on diverse facets of an individual’s life, including their career trajectory, interpersonal relationships, and various other aspects of daily life [5].

Insomnia is prevalent among adults with ADHD [6]. The relationship between ADHD and sleep problems is reciprocal, with ADHD symptoms potentially causing insomnia and improved sleep quality possibly alleviating ADHD symptoms. Factors such as side effects of ADHD medications, hyperactivity, and restlessness may contribute to sleep disturbances in individuals with ADHD [7]. Research suggests that bright light therapy in the morning has promise in improving sleep-related issues in adults with ADHD. Those dealing with both ADHD and insomnia must maintain good sleep hygiene, consider behavioral therapies, and consult a doctor for appropriate treatment options when needed.

Insomnia, an integral part of the mental health spectrum, is highly affected by stressful environmental events or the distressing, violent, and aggressive content of war, which has a global impact on mental health [8,9]. It has been reported that there is a link between the severity of ADHD symptoms, different dimensions of ADHD symptoms, symptoms of insomnia, and sleep duration in adults. This underscores the recurring connection between noteworthy ADHD symptoms, specifically inattention and hyperactivity, and insomnia symptoms, alongside changes in sleep duration. There is an urgent need to evaluate and address insomnia and changes in sleep duration among adults with ADHD [10]. Other studies have reported sleep-related factors in adults diagnosed with ADHD. They revealed that approximately 85% of participants experienced excessive daytime sleepiness or subpar sleep quality, with prevalent issues including difficulty falling asleep initially, disrupted sleep, and feeling excessively warm during sleep. Moreover, distinctions emerged between individuals who predominantly exhibited inattentive symptoms (ADHD-I) and those with combined symptoms (ADHD-C). The ADHD-I group reported lower sleep quality and increased fatigue compared to the ADHD-C group, with a notable interplay between subtype and gender influencing perceptions of fatigue [11].

The potential link between the use of sleep aids, specifically antihistamines, and the risk of ADHD in children is an evolving research topic. The impact of such aids on adult ADHD has not been adequately studied [12]. It is recognized that the relationship between sleep and ADHD is complex. Children with ADHD experiencing persistent sleep-onset insomnia may find melatonin beneficial in improving both the time taken to initiate sleep and overall duration of sleep. Sleep problems are common in adults with ADHD, and the interplay between ADHD and sleep disturbances is bidirectional [7]. While the precise effects of sleep aids on adult ADHD are not fully understood, it is crucial to consider potential consequences, including those of antihistamines, and seek guidance from healthcare professionals when addressing sleep issues in the context of ADHD [13].

The relationship between adult ADHD and sleep aid use has yet to be explored in Jordan. Nevertheless, existing research has investigated the impact of stimulant medications on the sleep patterns of adults with ADHD [7,12]. These investigations suggest that stimulant medications may induce side effects that lead to insomnia and compromise the overall quality of sleep in this population. Therefore, it is crucial for clinicians to monitor and address the potential impact of stimulant medications on sleep dynamics in adults with ADHD.

The principal objective of this study is to examine the potential correlation between self-administration of sleep aids and various clinical factors leading to heightened ADHD symptoms in a cohort of individuals in Jordan who underwent screening for insomnia during the ongoing war in Gaza. This study aims to address a significant void in the current academic literature by specifically focusing on the interconnection between self-medication practices involving sleep aids, coupled with other clinical variables, and the emergence of elevated ADHD symptoms. Existing studies have primarily explored ADHD symptoms and their associations with sleep-related challenges, as shown in the SWOT analysis in Table 1. However, there is a noteworthy deficiency in the research conducted in the Jordanian context, particularly concerning self-medication practices with sleep aids. Through this inquiry, we aim to provide innovative perspectives that not only enhance the prevailing comprehension of ADHD symptoms in the context of insomnia, but also furnish healthcare practitioners, policymakers, and researchers engaged with the Jordanian population with valuable insights. The study is meticulously crafted to bridge this gap and deliver a more comprehensive understanding of the complex relationship between self-medication practices, clinical factors, and ADHD symptoms, thereby contributing to the intellectual discourse in this specialized domain. The study’s characteristics are evaluated in Table 1 below.

## 2. Materials and Methods

### 2.1. Study Design and Recruitment

This cross-sectional study recruited a cohort of Jordanians using a convenient sampling method. The web-based study was approved by Yarmouk University IRB committee (protocol code 692) on 28 December 2023. All the participants read about and agreed to be enrolled in the study by choosing the option “I agree to participate” on the informed consent form provided by the corresponding author. All of the data obtained were anonymous. The study instrument was uploaded onto a Google Form, and the link was distributed on various social media platforms in Jordan. Data were collected during January 2024. The sample size was based on a confidence level of 95%, a confidence interval of 5%, and an estimated population size of 10 million. This resulted in the need to recruit 384 participants before the inclusion criteria could be applied.

### 2.2. Inclusion Crieria

Exclusive consideration was given to adults who reported clinically significant insomnia, as determined by the Arabic version of the Insomnia Severity Index (ISI-A) [14]. Developed by Morin et al. [15], the ISI-A consists of seven questions with Likert-type responses and produces a score in the range of 0 to 28. A cut-off score exceeding 14 is established as an indicative threshold for severe insomnia symptoms.

### 2.3. Study Instrument

#### Covariates

Demographic data and relevant information were systematically recorded, encompassing variables such as gender (male or female), age (below 30 years old or 30 years old and above), marital status (single or married), number of family members (fewer than five members or five members or more), the highest level of education completed (bachelor’s degree or graduate studies), smoking status (smoker or non-smoker), participants’ affiliation with the medical field (affiliated or unaffiliated), employment status (employed or unemployed), and any previous diagnoses of chronic conditions, with a primary focus on hypertension, diabetes, and dyslipidemia. To determine the specific self-medication practices related to sleep aids within the study sample, participants were given the autonomy to choose one or more options from the following categories: “homeopathy herbal remedies”, “over-the-counter sedating antihistamines”, or “never used any sleep aid”.

### 2.4. Outcome Variable

#### ADHD Symptom Severity

The assessment of symptoms resembling ADHD was carried out using the validated Arabic version of the Adult ADHD Self-Report Scale-V1.1 (ASRS), which comprises an 18-item scale. Aligned with the diagnostic criteria outlined in the Diagnostic and Statistical Manual of Mental Disorders (DSM) for ADHD, this scale produces a score in the range of 0 to 72, with higher scores indicating more severe symptoms [16,17].

### 2.5. Data Analysis

Frequencies and percentages were used to describe the demographics of the study sample. To determine which covariates are associated with ADHD severity, a preliminary univariate linear regression analysis was carried out, and potential confounders showing *p* < 0.1 were included in the multivariate linear regression analysis. Confidence intervals were set at 95%, and significance was set at *p* < 0.05. Data were analyzed using SPSS version 21.

## 3. Results

### 3.1. Response Rate

A total of 542 participants were approached, 487 agreed to participate, and 263 participants did not meet the inclusion criteria for insomnia; therefore, the data from 224 participants were analyzed.

### 3.2. Study Sample Demographics

The demographic analysis of the study resulted in insightful findings about the participant profile. Out of the initial 542 participants approached, 487 consented to participate, resulting in a comprehensive dataset of 224 participants after excluding 263 individuals who did not meet the inclusion criteria for insomnia. Among the participants, 156 (69.6%) were females, highlighting a significant gender disparity in favor of women. It is worth noting that 156 (69.6%) participants were single, indicating a substantial proportion of unmarried individuals in the study. From a demographic standpoint, 118 (52.7%) participants reported having five or more family members, a factor that warrants consideration due to its potential implications for sleep patterns and overall well-being. Additionally, the employment status of the participants was significant, with 155 (69.2%) reporting unemployment, which may have an impact on lifestyle and sleep routines. Regarding lifestyle factors, 90 (40.2%) participants disclosed being smokers, providing further insight into potential contributors to sleep patterns and overall health. Shifting the focus to the usage of sleep aids, 147 (65.3%) participants reported not using any sleep aids, indicating a prevalent reliance on natural sleep patterns. Among those who did use sleep aids, 50 (22.3%) opted for homeopathic remedies, revealing a preference for natural or alternative approaches to sleep management. Moreover, 41 (18.3%) participants reported using over-the-counter antihistamines as sleep aids, highlighting a segment of the population that relies on pharmaceutical options. These meticulous demographic details and sleep aid usage patterns present a nuanced portrayal of the study population, establishing a solid foundation for a comprehensive analysis of the correlation between sleep aid utilization and ADHD-like symptoms in adults grappling with insomnia. The detailed results are succinctly summarized in Table 2.

### 3.3. Correlates of ADHD Symptom Severity

The severity of ADHD symptoms was evaluated using the validated Arabic version of the ASRS. Higher scores indicated greater ADHD severity. To identify factors associated with ADHD, an initial univariate linear regression analysis was conducted (as shown in Table 3), followed by a comprehensive multivariate analysis with ADHD as the dependent variable (Table 4). It is worth noting that the final model was adjusted for both “age” and “diagnosis with chronic diseases”. The results of the analysis revealed a significant association between individuals aged above 31 years and lower ADHD severity (B = −3.95, t = −2.32, *p* = 0.002). Conversely, a diagnosis of chronic diseases was significantly associated with higher ADHD severity (B = 4.15, t = 1.99, *p* = 0.04), suggesting a notable correlation between health conditions and the manifestation of ADHD-like symptoms. Interestingly, the utilization of sleep aids did not demonstrate any statistically significant association with ADHD-like symptoms. Therefore, it can be inferred that the presence or absence of sleep aid usage did not impact the severity of ADHD symptoms in the study population. These findings, systematically presented in Table 3 and Table 4, contribute to a comprehensive understanding of the factors influencing ADHD severity in the cohort under study. They have implications for both clinical considerations and future research endeavors.

## 4. Discussion

The objective of this investigation was to examine the potential association between the use of sleep aids, such as antihistamines or homeopathic remedies, and ADHD-like symptoms in adults being screened for insomnia. Our findings indicate that the sleep aids used by the participants did not have any significant correlation with the severity of ADHD symptoms. However, we did find other factors that were strongly correlated with the severity of ADHD symptoms in the study population. Younger age was significantly associated with more severe ADHD symptoms, highlighting the importance of age in understanding the manifestation of ADHD-like symptoms. Additionally, individuals with chronic illnesses were also linked to higher ADHD symptom severity, suggesting a potential interaction between health conditions and the severity of ADHD symptoms in adults with insomnia. These findings provide valuable insights into the complex relationship between sleep aid usage, demographic factors, and the severity of ADHD-like symptoms. Further research is needed to fully explore the connections among these variables and gain a more comprehensive understanding of the factors influencing ADHD symptomatology in individuals undergoing insomnia screening.

The use of antihistamines is well established for dermatological and respiratory allergies. Previous studies have related the use of antihistamines to an increased risk of ADHD symptoms in children. For example, in one pilot retrospective study on children aged 6–12 years with atopic dermatitis, the study concluded that previous exposure to antihistamines was associated with about a two-fold incidence of developing ADHD symptoms [18]. In addition, another recent cohort study recruiting data from >40,000 children has demonstrated that children exposed to antihistamines have a 35% risk of ADHD [19]. Our findings revealed that antihistamines were not associated with ADHD symptom severity. Although the precise explanation of this finding requires additional larger-scale studies, several factors can provide insights. One possible explanation is that the metabolism and the distribution of these sedating antihistamines are different in adults, thus leading to alteration in the drug’s bioavailability and therefore its concentration in the site of action, in this case, the central nervous system. Another explanation is that the present study examines the whole class of antihistamines without stratification of each medication in this group. Perhaps future studies could study the effect of individual antihistamines such as chlorpheniramine or diphenhydramine that are frequently consumed as over-the-counter sleep aids [20]. Another explanation is that other demographics and clinical factors such as the chronic diseases of the participants and the chronically received medications could interfere with cognition. This is one of few studies that brings adult ADHD under the spotlight. Previous studies have indicated that the vast majority of adults with ADHD are underdiagnosed and undertreated [2]. The proper diagnosis of ADHD is quite challenging as it overlaps with other psychiatric illnesses, such as depression and anxiety [21], especially in developing countries such as Jordan where these disorders are stigmatized.

In the present study, it was found that participants aged above 30 years old were less likely to experience symptoms of severe ADHD and vice versa, i.e., participants with an age lower than 30 years were at a lower risk of developing severe ADHD symptoms. This supports previous research showing that a majority of adults with ADHD had symptoms in their youth [22]. The higher prevalence of ADHD symptoms in younger adults may be due to the challenges they face in educational settings, which are more demanding compared to the flexible working environments available to adults [23,24]. These findings emphasize the importance of considering developmental stages and environmental factors when studying ADHD symptoms in adults. Further research is needed to explore the relationship between age, environmental stressors, and ADHD symptoms for a more comprehensive understanding of this complex interaction.

The presence of chronic diseases in our cohort was predictive of higher ADHD symptom severity. In the present study, chronic diseases were mainly cardiovascular (hypertension and diabetes). This finding is consistent with previous studies. For example, a large cross-sectional study confirmed a positive association between cardiovascular disease and ADHD [25]. In addition, a recent study revealed that 46% of patients with type 2 diabetes reported ADHD-like symptoms [26]. This could be explained by the fact that subjects with ADHD-like symptoms could adopt negative behaviors that exacerbate metabolic control [27]. Moreover, both cardiovascular and cognitive impairment could share common ground in stress and inflammation [28,29]. Additionally, the medications used for chronic diseases could predispose patients to cognitive and mood disturbances [30,31]. The cross-talk between cardiovascular diseases and ADHD symptoms has common ground in biological backgrounds. This includes implication of the immune system, inflammatory cascades, neuromodulation, and hormonal dysregulation mainly in the hypothalamic–pituitary–adrenal (HPA) axis, as in [32]. In addition, the daily consumption of cardiovascular medications could predispose people to cognitive-related symptoms, although research in this area did not result in conclusive results [30,33]. This represents an attractive and challenging topic to investigate due to the complexity of the demographical, clinical, and patient’s intrinsic factors. For example, some studies could not relate the use of cardiovascular medications to impaired cognition [33]; on the other hand, another investigation that recruited a cohort of geriatric subjects concluded that the use of cardiovascular medications was significantly associated with lower incidence of cognitive impairment [34].

This study contributes to the limited literature focusing on adult ADHD symptoms. Although the idea, the validated scales, and the statistical model are all considered strengths, the study has some limitations. The symptoms of ADHD were not assessed by a professional psychiatrist. Although the study used a validated scale, as in previous studies, the self-reported scales could be associated with high prevalence rates compared to accurate medical or psychiatric diagnosis. Another limitation is that the study findings cannot be generalized to the Jordanian population as this would require an expansion of the study sample. In addition, the study did not examine other potential confounders, such as lifestyle, food and water consumption, the potential effect of chronic medication, and others. Also, the design did not include the names of specific antihistamines or herbal remedies. Furthermore, the cross-sectional design did not allow for the examination of the causal relationship between sleep aids and ADHD symptoms.

## 5. Conclusions

Our study investigated the correlation between the use of sleep aids and ADHD-like symptoms in adults with insomnia in Jordan. Among the 244 participants who had severe insomnia, 65.3% did not use any sleep aids. Homeopathic remedies were chosen by 22.3% of participants, while 18.3% used over-the-counter antihistamines. Contrary to our initial hypothesis, regression analysis did not find any statistically significant relationship between the use of sleep aids (specifically homeopathic herbal remedies and over-the-counter antihistamines) and ADHD-like symptoms (*p* > 0.05). However, certain demographic factors did affect the severity of ADHD symptoms. Participants being over the age of 31 showed a significant association with lower ADHD severity, while a diagnosis of chronic diseases was linked to higher ADHD severity. The analysis of demographic data provided intriguing insights into the study population. The higher prevalence of females and unmarried individuals suggests that gender and marital status may influence the severity of ADHD. Furthermore, information on family size, employment status, and smoking habits helped us gain a better understanding of the cohort. Correlational analysis, including univariate and multivariate linear regression, further supported the impact of age and chronic diseases on ADHD severity. These findings underscore the importance of considering both demographic and health-related factors when evaluating adult ADHD. Importantly, the use of sleep aids did not contribute to ADHD-like symptoms in this particular population. These results contribute to the existing research on adult ADHD and emphasize the significance of age and health status in determining the severity of symptoms. Future studies should explore the intricate relationship between insomnia, sleep aids, and ADHD in diverse populations to gain further insights into potential risk factors and approaches to treatment.

## Figures and Tables

**Table 1:** Evaluation of study characteristics—strengths, weaknesses, opportunities, and threats in investigating the relationship between sleep aids and ADHD in the Jordanian population.

**Strengths**	**Weaknesses**	**Opportunities**	**Threats**
- Addresses a specific gap in the literature by focusing on the Jordanian population, providing a unique and context-specific perspective on the relationship between sleep aids and ADHD.	- Limited Generalizability: Focus on the Jordanian population may restrict applicability to other cultural or demographic contexts.	- Informing Interventions: Positive findings could contribute to targeted interventions for Jordanian individuals experiencing ADHD symptoms in the context of insomnia.	- External Factors: Economic, political, or social factors in Jordan may impact study implementation and outcomes.
- Comprehensive approach: Aims to explore the multifaceted relationship by considering various clinical factors, contributing to a more thorough understanding of the subject.	- Potential Bias: Self-reporting of sleep aid use and ADHD symptoms may introduce bias as participants may not accurately recall or report their behaviors.	- Guidance for Healthcare Practices: Study can guide healthcare practitioners in addressing self-medication practices with sleep aids and managing ADHD symptoms.	- Limited Participation: Difficulty in recruiting a representative sample may compromise study validity and applicability.
- Practical Implications: Findings could have practical applications for healthcare practitioners, policymakers, and researchers, offering valuable insights for potential interventions or guidelines.	- Complexity of Variables: Involvement of various clinical factors may introduce complexity, making it challenging to isolate the direct impact of sleep aids on ADHD symptoms.	- Foundation for Further Research: Successful completion could lay the groundwork for further research exploring similar relationships in diverse populations or refining methodologies.	- Ethical Considerations: Ensuring participant confidentiality and addressing potential ethical concerns related to self-medication practices and mental health disclosures is crucial.

**Table 2:** Sample characteristics (*n* = 224).

Factor	Category	*n* (%)
Sex	Male	68 (30.4)
	Female	156 (69.6)
Age	Below 30 years	148 (66.1)
	31 years and above	76 (33.9)
Marital status	Single	156 (69.6)
	Married	68 (30.4)
Family members	Fewer than 5	106 (47.3)
	5 or more	118 (52.7)
Highest education	Bachelor’s	190 (84.8)
	Graduate studies	34 (15.2)
Employment status	Unemployed	155 (69.2)
	Employed	69 (30.8)
Are you studying?	no	131 (58.5)
	yes	93 (41.5)
Are you in the medical field?	No	106 (47.3)
	Yes	118 (52.7)
Smoking status	Non-smoker	134 (59.8)
	Smoker	90(40.2)
Diagnosed with chronic diseases?	No	183 (81.7)
	Yes	41 (18.3)
I use herbal homeopathy preparations for sleep		50 (22.3)
I use over-the-counter antihistamines for sleep (sedating antihistamines)		41 (18.3)
I do not use any sleep aids		147 (65.3)

**Table 3:** Univariate linear regression for ADHD symptoms as the dependent variable.

Factor	B	t	*p*	95% CI
Female gender	2.67	1.51	0.13	−0.80–6.13
Age above 31 years	−3.53	−2.08	0.03 *	−6.88–−0.19
Married	−3.89	−2.29	0.02 *	−7.33–−0.45
Five or more family members	1.54	0.95	0.34	−1.66–4.74
Graduate studies	−2.64	−1.17	0.24	−7.09–1.80
Employed	0.47	0.26	0.79	3.0–3.93
Student	1.00	0.61	0.54	−2.24–4.26
Medical field	2.67	1.65	0.10	−0.51–5.86
Smoking	2.99	1.81	0.07	−0.25–6.23
Diagnosed with chronic diseases	3.55	1.70	0.09	−0.56–7.67
I use herbal homeopathy preparations for sleep	−1.32	−0.68	0.49	−5.16–2.51
I use over-the-counter antihistamines for sleep	1.37	0.65	0.51	−2.76–5.51
I do not use any sleep aids	−0.53	−0.31	0.75	−3.91–2.83

The ADHD symptom severity was assessed using the validated Arabic version of ASRS. B: beta, t: t-value, CI: confidence interval, * *p* < 0.05.

**Table 4:** Multivariate linear regression for ADHD symptom severity as the dependent variable.

Factor	B	t	*p*	95% CI
Age above 31 years	−3.95	−2.32	0.002	−7.31–−0.60
Diagnosis with a chronic disease	4.15	1.99	0.04	0.05–8.26

The ADHD symptom severity was assessed using the validated Arabic version of ASRS. CI: confidence interval.

## Data Availability

Data associated with this publication will be available from the corresponding author upon request.
